# Supplementary material for: Fiber architecture in the human ventromedial striatum and its relation with the bed nucleus of the stria terminalis
Source: PLoS One. 2025 May 7;20(5):e0323113. doi: 10.1371/journal.pone.0323113 (PMC12057886; doi:10.1371/journal.pone.0323113)
Supplement: Attachment — Submitted filename: Response to Reviewers.docx [file pone.0323113.s003.docx]

Diagnostic and Interventional Neuroradiology

Department Radiology • Diagnostic and Interventional Neuroradiology

Hoppe-Seyler-Str. 3 • 72076 Tübingen • Germany

Head of Neuroradiology

Prof. Dr. U. Ernemann

**Revision PONE-D-24-36525**

24.03.2025

Dear editors, dear reviewers,

thank you for providing us with the opportunity to revise and resubmit our manuscript titled “Fiber architecture in the human ventromedial striatum and its relation with the bed nucleus of the stria terminalis” (PONE-D-24-36525). In this letter we would like to address all concerns raised about our initial draft.

Responses to points raised by the editor:

1. We reformatted the manuscript to meet formatting requirements.
2. The funders had no role in study design, data collection and analysis, decision to publish, or preparation of the manuscript.
3. All authors declare no competing financial interests or potential conflicts of interest in relation to the work described. Outside of the submitted work BB has received consultancies from Medtronic (paid to institution) and is co-founder of AIRAmed GmbH. This does not alter our adherence to PLOS ONE policies on sharing data and materials.
4. We have made all data used for this study available for download via data repositories. Please update our Data Availability statement as follows: “Raw data of polarized light microscopy images and background images for correction as well as a compiled tool for the evaluation of polarized light microscopy images in the way used in this study are available for download under doi: 10.12751/g-node.xl2ctk.
   14T MRI data of the tissue samples, anonymized 3T MRI data of the study participants, and masks underlying the analysis depicted in Figure 1 are available under doi: 10.12751/g-node.1lfvzi.”
   In addition, the single FA values extracted from the abovementioned masks are now provided as supporting data in text format (FA_data.zip) with one file for each extracted segment of each participant (Sub_*no*_Seg_*no*_FA), one file for each participant containing mean FA values for the six different segments (Sub_*no*_mean_FA), as well as one file with above participant averages for the six different segments (Mean_FA_values).
5. To the best of our knowledge, our reference list does not contain articles that have been retracted.

Responses to points raised by reviewer 1:

1. We added a paragraph on the use of polarized light microscopy in medical applications to our introduction.
2. Polarization filters were introduced into the light path with an individually in-house manufactured holder. The sentence on the placement of the different filters was reworded for better understandability.
3. We have added additional information to the caption of Fig 4.
4. The individually extracted and segmented ROIs from our participants’ FA maps underlying the values displayed in Fig 1 are now part of our data repository under doi: 10.12751/g-node.1lfvzi. The extracted FA values for each participant and segment have furthermore been uploaded as supporting data.

Responses to points raised by reviewer 2:

We acknowledge that the extent of myelination of nerve fibers in the brain plays an important role in the integrity of signaling pathways, especially in the light of recent insights on the role of myelin sheaths in the plasmon-polariton model of signal transduction. Unfortunately, the technique we used in the study at hand is limited in this regard. We have dedicated an additional segment to address these limitations in our paragraph on Methodical considerations.

In addition to the matters above we would like to point out that during data curation we noticed some inconsistencies in our calculations underlying figure 1. We corrected the figure and corresponding text passages accordingly.

With kind regards

Dr. Oliver Krüger
